# Modifying macronutrients is superior to microbiome transplantation in treating nonalcoholic fatty liver disease

**DOI:** 10.1080/19490976.2020.1792256

**Published:** 2020-08-20

**Authors:** Fontini Tania Mitsinikos, Denise Chac, Nicholas Schillingford, R. William DePaolo

**Affiliations:** a Department of Gastroenterology, Hepatology and Nutrition, Children’s Hospital of Los Angeles, Los Angeles, CA, USA; b Department of Pathology, University of Washington, Seattle, WA, USA; c Department of Medicine, University of Washington, Seattle, WA, USA; d Center for Microbiome Sciences & Therapeutics, Seattle, WA, USA

**Keywords:** Liver, microbiome, nutrition, NAFLD

## Abstract

Nonalcoholic fatty liver disease (NAFLD) is the leading cause of chronic liver injury and liver transplantation in Western countries. The pathogenesis of NAFLD includes overnutrition-associated metabolic syndrome or the improper consumption of dietary macro- and micro-nutrients that either support or prevent disease development. This altered nutrient landscape has been linked to shifts within the gut microbiota which can exacerbate liver pathology and the progression of NAFLD. Treatment goals for NAFLD target lifestyle and dietary modifications that restrict calories and adjust macronutrient content. It is not well understood how different macronutrients alter the microbiota and whether the diet-educated microbiota contribute to the resolution of disease. We fed mice a diet high in fat, cholesterol and fructose for 6 weeks and then in two different arms of the study, intervened with either a diet high in saturated and polyunsaturated fats and fiber or low in fats and fiber. In a second set of experiments, we performed microbiota transplants using cecal contents from mice fed one of the intervention diets to assess whether the diet-educated microbiota could impact clinical outcomes in mice fed a NAFLD-inducing diet. Pathology, steatosis, ALT/AST levels, and liver cytokine levels were measured as primary outcomes. We found that despite different microbiota compositions, both of the intervention diets reversed the progression of NAFLD and dampened inflammation. In contrast, transplantation of cecal contents from the intervention diet-fed mice to mice receiving a NAFLD-inducing diet was unable to prevent disease progression, and, in some cases, worsened disease. These data underscore the importance of dietary modifications to treat NAFLD and caution against the use of microbiota transplantation in the absence of dietary and lifestyle modifications.

## Introduction

Nonalcoholic fatty liver disease (NAFLD) is the most common cause of liver disease in the Western world affecting 80–100 million adults and children in the United States and an estimated 1 billion people worldwide.^1^ NAFLD refers to a spectrum of liver diseases that range from bland steatosis to nonalcoholic steatohepatitis (NASH). NASH is the progressive form of NAFLD and, if left untreated, can lead to fibrosis, cirrhosis and hepatocellular carcinoma. The pathophysiology of NAFLD is not entirely understood. Though it typically occurs in the context of obesity or metabolic dysregulation, disease is thought to be multifactorial including diet, environment and the gut microbiome.^2,3^

The importance of the intestinal microbiota in obesity and NAFLD development has been shown using germ-free (GF) mice. GF mice are resistant to diet-induced obesity^4^ and colonization with the microbiota from conventionally raised or specific pathogen-free mice increases body fat and insulin resistance.^5^ The current paradigm suggests that the increased consumption of dietary fat and fructose alters the gut microbiota.^6^ Production of potentially harmful molecules by this altered microbiota can be absorbed in the intestine and carried to the liver, causing a chronic low-grade inflammatory state.^6^ However, the molecular mechanisms contributing to NAFLD by the gut microbiota are likely complex and may include the regulation of energy homeostasis,^5,7^ synthesis of triglycerides,^8,9^ lipoprotein synthesis,^10^ bile acid homeostasis^11,12^ and bacterial-derived toxins or virulence factors.

Currently, the most effective treatment for NAFLD consists of modifying dietary intake of fat and sugar and increasing physical activity.^13^ Diet also has a strong influence on the composition and function of the microbiota.^14-17^ According to international guidelines limiting the intake of calories, saturated and trans fatty acids, and fructose, while increasing the intake of lean protein, fibers, and omega-3 polyunsaturated fatty acids (PUFA) are the dietary modifications necessary to treat NAFLD.^18^ Altering the protein and fat content, the carbohydrate load or the presence of specific bioactive compounds including omega-3 PUFAs and fibers can influence the intestinal microbiota by altering bacterial diversity or through regulating the metabolites they produce.^19^ Despite the obvious connections between macronutrients in the diet, the microbiome and NAFLD, there is a paucity of studies looking at this relationship.

Using diets high in mono- and polyunsaturated fats and fiber or low in fat and fiber, we evaluated their impact on the progression of NAFLD and the effects of these interventions on the microbiota. Further, we sought to dissociate the effects of the diet-educated microbiota from the dietary intervention using microbiota transfers. Our results indicate that both intervention diets were able to reverse hepatic steatosis and were accompanied by distinct changes in the microbiota. However, the microbiota was unable to transfer this phenotype and actually accelerated fibrosis when given to mice concomitantly receiving food high in cholesterol and fructose. These data suggest that diets modified in types of fats and fibers can be efficacious at reversing the progression of NAFLD but cautions that manipulating the microbiota of patients with NAFLD or NASH may not be effective without continued dietary changes.

## Results

### A rodent diet high in fat, cholesterol and fructose mimics liver pathology of human NAFLD

**Figure 1.**
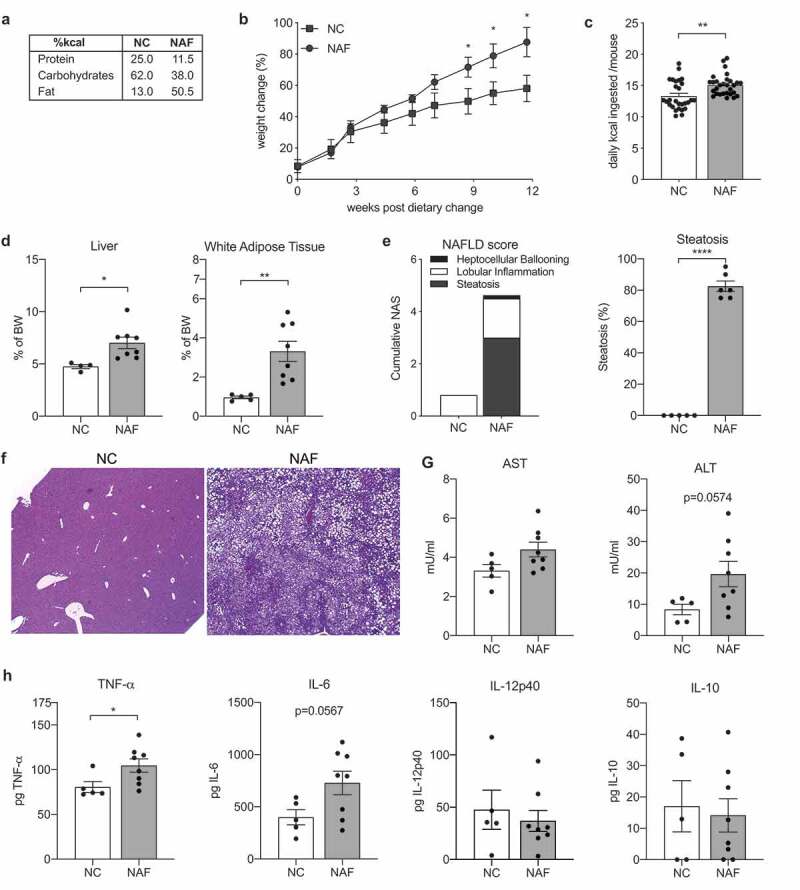
Liver inflammation and steatosis induced by a diet high in fructose, cholesterol and low in fiber. C57Bl/6 mice were treated with a nonalcoholic fatty liver disease diet (**NAF**) or normal chow (**NC**) for 12 weeks. (a) macronutrients of diets. (b) Percent weight change during diet. (c) Daily consumption by kilocalories (kcal) per mouse per diet. (d) Liver and white adipose tissue weights by percent body weight. (e) NAFLD-activity score (NAS) and percent steatosis of liver. (f) Representative images of hematoxylin & eosin (H&E) and trichrome stained liver sections. (g) Aspartate transaminase (AST) and alanine transaminase (ALT) levels measured in serum. (h) Levels of TNF-a, IL-6, IL-12p40, and IL-10 cytokine in homogenized liver tissue. Data is the mean ± SEM of 2 independent experiments, n = 5–8 mice/group. Statistics for (B) is 2-way ANOVA, Sidak’s multiple comparisons; (C-E, G-H) is Student’s unpaired t-test. *, *p* < .05; **, *p* < .01; and ****, *p* < .001.

There are a number of animal models that recapitulate the various hallmarks of NAFLD using deficient diets such as the methionine- and choline-deficient diet and the high fructose diet.^20,21^ However, these diets, have additional changes not related to the human counterpart including altered metabolic profile^22^ and disparate fat accumulation in the liver.^21^ In this study, we developed a NAFLD-inducing diet (NAF) consisting of high fat, cholesterol and fructose. To establish the modified diet’s ability to induce steatosis and nonalcoholic steatohepatitis (NASH), C57Bl/6 mice were fed either a control normal-chow diet (NC) or NAF for 12 weeks (Figure 1(a)). Mice on NAF gained more weight than NC mice at week 9 through to week 12 (Figure 1(b)) and this correlated with an increase in daily consumption of kilocalories (Figure 1(c)). After 12 weeks, mice fed the NAF diet had heavier livers and white adipose tissue than NC fed mice (Figure 1(d)). Development of NAFLD was evaluated through liver histology. As expected, NAF mice had a high NAFLD Activity Score (NAS) with high scores for steatosis, lobular inflammation and presence of hepatocellular ballooning (Figure 1(e)). NAF fed mice also had more steatosis with 80% fatty liver accumulation. Histology also revealed both micro- and macro-vesicular fat accumulation with greater steatosis (figure 1(f)) and mild fibrosis (Supplemental Figure 1) in the NAF-fed group.

One hallmark of the progression of NAFLD to NASH is the increase of inflammatory associated markers.^23^ Liver enzymes aspartate aminotransferase (AST) and alanine aminotransferase (ALT) were measured in serum and cytokines were measured in liver homogenates. Despite elevated levels of both AST and ALT in NAF mice, they did not reach statistical significance (Figure 1(g)). However, mice on NAF did have statistically elevated levels of liver TNF-α and nearly significant levels of IL-6, but no difference in IL-12p40 or IL-10 (Figure 1(h)). Taken together, these data indicate that a diet high in fructose, cholesterol and fat induces a disease similar to human NAFLD with increased steatosis, liver inflammation, and elevated AST and ALT levels.

### Dietary intervention suppresses weight gain and reverses fat accumulation in the liver

**Figure 2.**
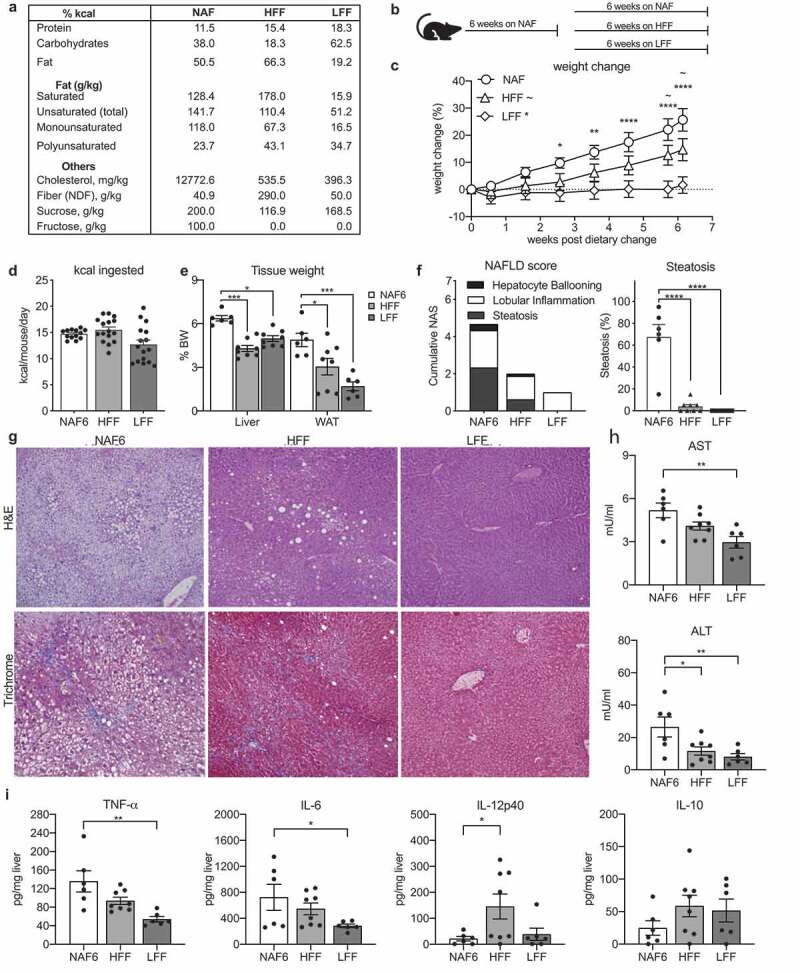
Both a high fat/high fiber and a low fat/low fiber diet can reverse the progression of NAFLD. C57bl/6 wildtype mice were treated with NAF for 6 weeks ad libitum and then introduced to a dietary intervention of either a high-fat/high-fiber diet (**HFF**) or a low-fat/low-fiber diet (**LFF**) for 8 additional weeks. (a) Macronutrient breakdown of diets and (b) model of dietary intervention. (c) Percent weight change during dietary intervention using NAF at 6 weeks (NAF6) as a baseline. (d) Daily consumption of diet by kilocalories (kcal) per mouse per diet. **E)** Liver and white adipose tissue weights by percent body weight. (f) NAS and percent steatosis of mice scored by a pediatric liver pathologist blinded to the samples. (g) Representative image of H&E and trichrome stained liver section. (h) AST and ALT levels measured in serum by ELISA. (i) Levels of TNF-a, IL-6, IL-12p40, and IL-10 cytokine measurements in 50 mg homogenized liver tissue. Data is the mean ± SEM of 2 independent experiments, n = 5–8mice/group. Statistics for (C) is 2way ANOVA, Dunnett’s multiple comparisons; (C-E, G-H) is one-way ANOVA, Dunnett’s multiple comparisons; *, *p* < .05; **, *p* < .01; ***, *p* < .005; and ****, *p* < .001.

Treatment for NAFLD through dietary modification aims to reduce weight, improve blood pressure and cholesterol levels, and reduce liver steatosis.^24,25^ In this study, we assessed the potential of preventing NAFLD using two different intervention diets. The first was modeled after a modern Paleolithic diet which is high in polyunsaturated fats, saturated fats from alternative sources, such as coconut oil and beef tallow, and high in fiber (HFF). The second intervention diet was modeled after as traditional low-fat diet reflecting the low amount of fiber that is consumed by most Americans (LFF) (Figure 2(a); Supplemental Table 1).

Mice on NAF for 6 weeks (NAF6) were switched to one of the intervention diets or maintained on NAF diet for an additional 6 weeks (NAF) (Figure 2(b)). By the end of the 6 weeks, the mice receiving the HFF diet had gained an average of 12%, while the LFF fed mice gained only 3% of their weight at the time of intervention. Both of these were significantly lower than the mice continuing on the NAFLD diet which had gained, on average nearly 25% of their weight at the beginning of the intervention (Figure 2(c)). The decreased weight gain observed in the intervention diets could be due to the consumption of less kilocalories and not the macronutrient content. The amount of kilocalories consumed per day after the dietary intervention was calculated. The NAF and HFF mice consumed similar amounts of kilocalories despite significantly less weight gain in the HFF-fed mice (Figure 2(d)). The mice fed the LFF diet consumed less kcal on average compared to the NAF and HFF-fed mice, however, it was not statistically significant (Figure 2(d)). Both dietary interventions reduced the weight of the liver and adipose tissue. However, the HFF had a more significant impact on liver weight while the LFF promoted loss of white adipose tissue (Figure 2(e)). To investigate whether the intervention could reverse or halt the progression of NAFLD, liver pathology of mice receiving the dietary interventions were compared to NAF6 mice. Both intervention diets lowered NAS scores and had significant reduction in lobular inflammation (Figure 2(f)). However, mice receiving the LFF diet had a complete reversal of steatosis and hepatocyte ballooning, while mice fed HFF still had minor hepatocyte ballooning and some steatosis (Figure 2(f)). The presence of fibrosis was assessed with Masson trichrome staining and demonstrated rare cases of stage I, focal subcapsular areas of steatosis, and instances of perisinusoidal fibrosis in the NAF6 mice while mice on the HFF or LFF diets had no observable fibrotic tissue (Figure 2(g)). Taken together, these data demonstrate that despite being high in saturated fats and having a similar kcal consumption to the mice fed the NAF diet, HFF-fed mice showed improvements in liver histology, steatosis and fibrosis, while the LFF-fed mice had complete resolution of liver pathology.

### Dietary intervention improves liver inflammation

Inflammation was assessed using serum levels of ALT and AST, which were elevated in mice given the NAF diet for 6 weeks (Figure 2(g)). The HFF diet significantly reduced levels of ALT, while both ALT and AST were lowered in the LFF-fed mice (Figure 2(g)). Analysis of cytokine levels using liver homogenates also revealed differences between the two intervention diets. Compared to NAF6 mice, the LFF diet significantly lowered levels of TNF-α and IL-6 (Figure 2(h)). In contrast, the HFF diet had little impact on liver levels of TNF-α or IL-6, but caused significantly higher levels of the proinflammatory cytokine, IL-12p40 (Figure 2(h)). Interestingly, levels of anti-inflammatory IL-10 trended higher in mice given either the LFF or HFF diet compared to mice receiving NAF diet for 6 weeks, but these levels did not reach statistical significance (Figure 2(h)). These data align with the histological improvements and suggest that implementing a dietary intervention high in saturated fats and fiber and can reverse hepatic steatosis and reduce inflammation in the liver but not as completely as a diet low in fat.

### Fecal microbial communities shift following dietary interventions

**Figure 3.**
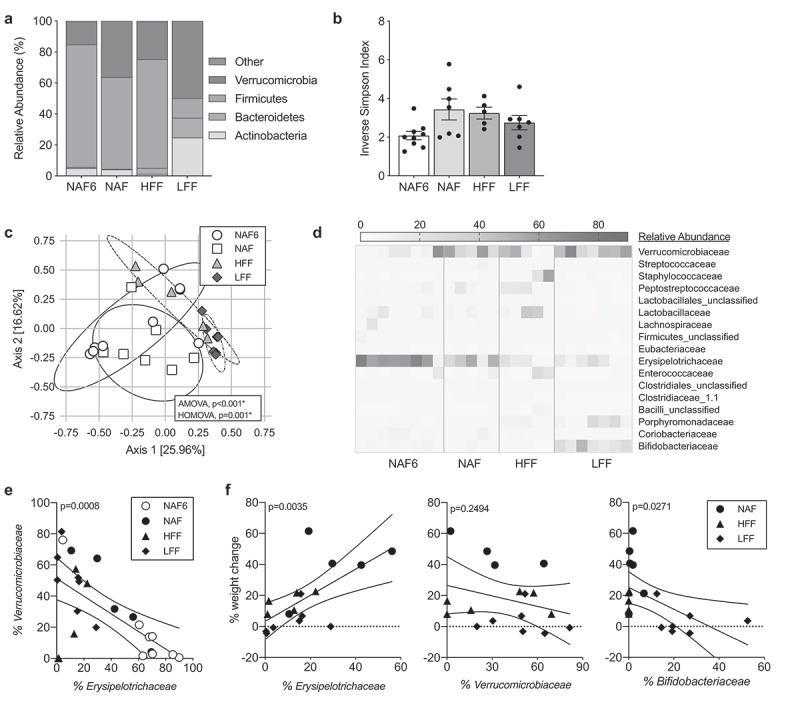
HFF and LFF diets cause distinct shifts in the composition of the microbiota following dietary intervention. Microbiota sampling of fecal contents of mice in treatment groups NAF6, NAF, HFF and LFF. (a) Phylum-level abundance and (b) diversity measured using Inverse Simpson Index. (c) Principal Coordinate of Analysis (PCoA) of microbiota composition at the genus level. (d) Heatmap representation of family-level abundance with families >1% abundance. (e) Relative abundance of *Erysipelotrichaceae* and *Verrucomicrobiaceae* by scatter plot (*p* = .0008). (f) Correlation between weight change and abundance of *Erysipelotrichaceae* (*p* = .0035), *Verrucomicrobiaceae* (*p* = .2494) and *Bifidobacteriaceae* (*p* = .0271). Data is the mean ± SEM of 2 independent experiments, n = 5–8mice/group. Statistics in (C) is analysis of molecular variance (AMOVA) and homogeneity of molecular variance (HOMOVA). (e-f) is Pearson Correlation Coefficient of all data points.

Diet has a major influence in shaping the gut microbiome, thus it is not surprising that changes in the composition of the microbiota have been associated with NAFLD. In order to assess shifts in the microbiota accompanying the resolution of steatosis in mice fed the intervention diets, we performed 16 s rRNA sequencing on ileal and fecal samples. After being on NAF for 6 and 12 weeks, there was a shift from a Bacteroidetes-dominant to a Firmicutes-dominant microbiome in the feces, as seen in other studies using high-fat diets (Figure 3(a); Supplemental Figure 2).^14^ HFF-fed mice had a similar phylum-level microbiota as NAF mice despite resolving steatosis and improving pathology (Figure 3(a)). In contrast, mice receiving the LFF diet had a significant increase in Actinobacteria and Bacteroidetes (Figure 3(a)). Analysis of the ileal microbiome revealed similar trends to those we had observed in the feces (Supplemental Figure 3A-B).

When comparing diversity using the Inverse Simpson Index at the genus level, we found that HFF-, LFF- and NAF-fed mice had a trend with more diversity than the NAF6 mice, however none reached statistical significance (Figure 3(b)). Despite a similar phylum-level composition, HFF mice had an increase in diversity compared to the NAF6 mice, suggesting differences at the genus level within Firmicutes. This was confirmed using principal coordinate of analysis (PCoA) using the Theta YC distances and showed an overlap in bacterial communities between the NAF6 and NAF that is significantly distinct from the microbiota of both the HFF and LFF via AMOVA (Figure 3(c)).^26^ The HFF community had a widespread cluster while the LFF-associated microbiota are tightly clustered with little overlap to NAF6 or NAF mice with statistical significance via HOMOVA (Figure 3(c)). PCoAs were also performed using both weighted and unweighted UNIFRAC distances and confirmed the data using Theta YC distances (Supplemental Figure 4). There was no difference between NAF6 and NAF groups. Using the weighted and unweighted UNIFRAC, we also saw statistical significance when comparing NAF to HFF, NAF to LFF and HFF to LFF (Supplemental Figure 4). To identify the differences in the microbiota communities, we visualized microbial families having greater than 1% abundance using a heatmap. Both NAF groups had high levels of *Erysipelotrichaceae* and *Verrucomicrobiaceae*, which made up over half of the microbiota composition (Figure 3(d)). Mice receiving HFF also had high abundance of *Erysipelotrichaceae*, but had increases in the abundance of Firmicutes families *Peptostreptococcaceae, Staphylococcaceae* and *Lactobacillaceae* with a decrease in *Verrucomicrobiaceae. Erysipelotrichaceae* was also abundant in LFF mice, but was accompanied by increases in *Porphyromonadaceae, Bifidobacteriaceae* and *Verrucomicrobiaceae*. Interestingly, the abundance of *Erysipelotrichaceae and Verrucomicrobiaceae* has an inverse relationship with each other (Figure 3(e), Supplemental Table 2). *Erysipelotrichaceae* also had a significant positive correlation with weight gain regardless of diet, while *Verrucomicrobia* trended with less weight gain (Figure 3(f), Supplemental Table 3). *Bifidobacteriaceae*, on the other hand, had a significant negative relationship with weight change regardless of diet (Figure 3(f)). These data demonstrate that despite resolution of steatosis by both dietary interventions each had a distinct microbiota profile from one another as well as from mice on the NAF diet.

### Microbiota transplantation fails to resolve steatosis and liver inflammation

**Figure 4.**
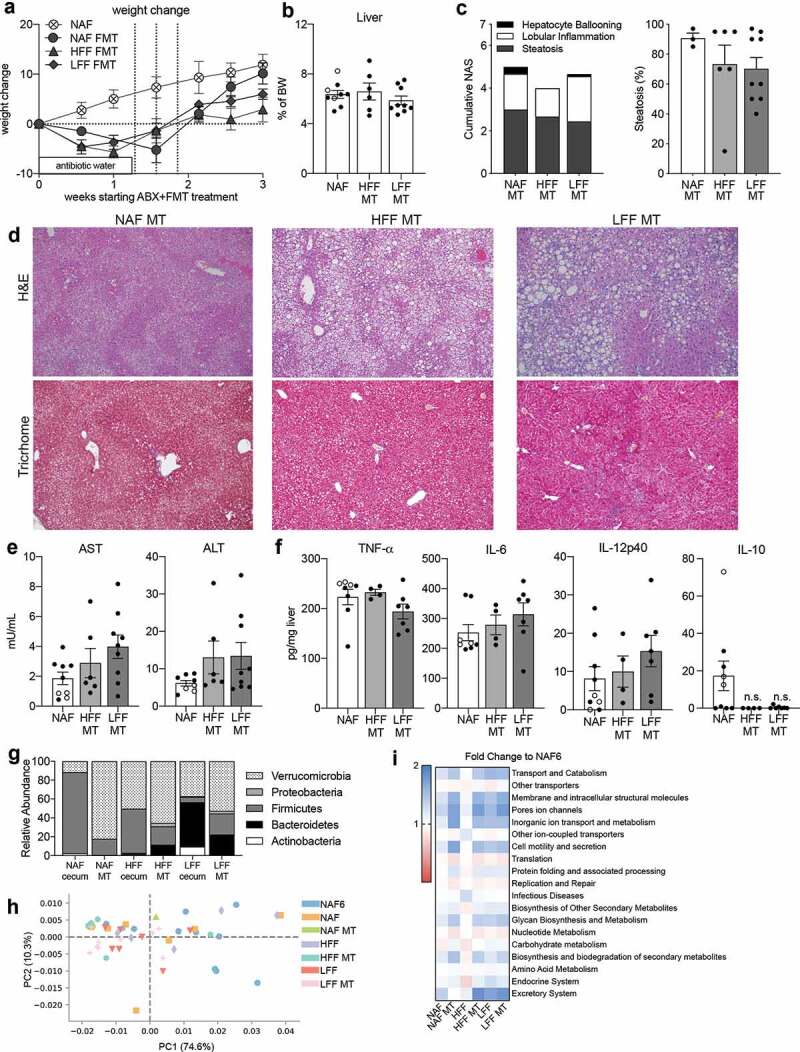
Diet-educated microbiota alone is not sufficient to reverse NAFLD. Mice on NAF for 6 weeks were given antibiotic water for 1–1.5 weeks and then given microbiota transplants (MT) using cecal contents from mice at the end of the diet intervention model. The recipient mice were then maintained on NAF for 2 weeks. (a) Percent weight change during MT using NAF at 6 weeks as baseline. (b) Liver weight as percent body weight. (c) NAS and percent steatosis of liver. (d) H&E and trichrome staining of liver histology. (e) AST and ALT measured in serum. (f) TNF-a, IL-6, IL-12p40, and IL-10 cytokine measurements of homogenized liver tissue. (g) Phylum-level abundance of donor cecal and treated fecal microbiota. (h) Principal component analysis and (i) heatmap of fold change of Phylogenetic Investigation of Communities by Reconstruction of Unobserved States (PICRUSt) analysis. Data is the mean ± SEM of 2 independent experiments, n = 3–9mice/group.

To understand whether the intervention diet-educated microbiota could reverse steatosis or inflammation independent of the intervention diet itself, we performed microbiota reconstitution experiments. Mice fed NAF for 6 weeks were placed on a cocktail of antibiotics in their drinking water for 10 days. At the end of this period the recipient mice were administered the cecal contents from mice that had previously undergone one of the dietary interventions or stayed on NAF. Post-reconstitution, the mice were maintained on NAF diet for an additional week. Microbiota depletion resulted in a 4–6 log reduction in bacteria as measured by copy number of the 16 S gene (Supplemental Figure 4A) and less than 10% weight loss was observed (Figure 4(a)). After the microbiota was reconstituted by oral gavage, mice receiving the microbiota from either HFF (HFF-MT) or LFF (LFF-MT) donors had less weight gain compared to mice receiving the microbiota from NAF donors (NAF-MT) or untreated NAF mice (Figure 4(a)). Liver (Figure 4(b)) and adipose tissue (Supplemental Figure 4B) weights were measured and no significant changes were observed.

Assessment of the liver pathology from NAF-MT mice displayed histological characteristics of late stage NAFLD/early NASH (Figure 4(c)). In addition to the presence of both macro- and microsteatosis, necroinflammatory foci and fibrosis were revealed by Masson trichrome staining (Figure 4(d)). Despite the decrease in weight observed in mice receiving cecal contents from either of the intervention diets, there were no histological improvements. Mice receiving cecal contents from either LFF or HFF diet had histological lesions that resembled the pathology of the NAF-MT and NAF-fed mice with similar overall NAS scores and a similar percentage of steatosis (Figure 4(c–d).

As expected from the amount of injury observed in the livers of mice receiving cecal contents from intervention diet mice, AST and ALT levels in the serum were as high as mice receiving the NAF diet (Figure 4(e)). In addition to the elevated AST and ALT, mice reconstituted with either intervention diet-educated microbiota had high levels of TNF-α, IL-6 and IL-12p40 in the liver (Figure 4(f)). Interestingly, IL-10 was not detected in the livers of the LFF-MT or HFF-MT mice but was present in the NAF-MT mice (Figure 4(f)). These data suggest that the microbiota from mice receiving dietary interventions, and who showed complete reversal of NAFLD, was unable to resolve disease in the absence of the dietary factors that shaped the microbiota. To determine if the transferred gut microbiota was maintained while on the NAF diet for 1 week following the last gavage, 16 S sequencing was performed on representative donor cecum samples and fecal samples post-microbiota transplant. Each of the recipient mice had a bacterial profile that differed from their respective donor. The NAF-MT mice had much more Verrucomicrobia, the HFF-MT mice had more Bacteroidetes and Proteobacteria and the LFF-MT mice had less Actinobacteria, but an increase in the abundance of Firmicutes compared to their respective donors (Figure 4(g)). When comparing the bacteria across the MT groups, we found no significant differences at the phylum or family levels (Figure 4(g), Supplemental Figures 5 & 6). To further understand why the microbiota of the intervention diets was unable to transfer protection from NAFLD, we compared the predicted metabolic functions of dietary intervention and MT mice using Phylogenetic Investigation of Communities by Reconstruction of Unobserved States (PICRUSt).^27^ Overall, there were significant differences in PICRUSt metabolic functions among mice on NAF for 6 weeks, the two dietary interventions and the MT groups (Figure 4(h)). To analyze specific changes between groups, each experimental group was compared to NAF6. While both LFF and the LFF-MT group had significant differences compared to NAF6, some of the metabolic features were higher in one, but not the other (Figure 4(h–i)). Interestingly, many of the identified metabolic pathways differed between diet group and its corresponding MT group. These data would suggest that the metabolic pathways of bacteria given during a microbial transplant are heavily influenced by the diet of the recipient.

In all, we show that administering a microbiota transplant without altering the diet does not improve NAFLD and is far less superior than dietary intervention in resolving steatosis. Whereas the dietary intervention significantly altered the microbiota, the NAFLD-inducing diet prevents changes in the microbiota despite MT and these changes are separate from predicted metabolic functions.

## Discussion

In this study, we demonstrate that altering specific macronutrients can be highly effective at reversing the progression of NAFLD in mice previously receiving a diet high in cholesterol, carbohydrates and fructose. These data demonstrate a reduction in steatosis, inflammation and liver enzyme levels following either of the dietary interventions. However, it is important to note that each diet had distinct effects on clinical characteristics and the microbiome. In contrast, microbiota transplants using cecal material from mice fed either of the intervention diets showed no clinical improvements and had liver pathology with inflammation and fibrosis. These results highlight the importance of diet in the treatment of NAFLD and further suggest that the use of microbiota transplants to treat NAFLD may not be efficacious in the absence of dietary and lifestyle changes.

Using a modern Western diet low in fiber, high in cholesterol and fructose, and a carbohydrate to fat ratio at 1–1.5, we sought to determine whether diets with specific nutritional modifications in fats, fiber and carbohydrates could halt the progression of NAFLD. Both of the dietary interventions were able to improve NAFLD symptoms but differed in their impact on liver enzymes and steatosis. While LFF completely reversed steatosis and significantly decreased both AST and ALT, HFF only partially resolved steatosis and only lowered AST.

While many studies show resolution of steatosis through a reduction in energy consumption, dietary factors such as the different macronutrients have also been shown to directly influence steatosis and the development of NAFLD. In a systematic meta-analysis of NAFLD studies, omega-3 supplementation was shown to decrease liver fat,^28^ while increasing MUFAs reduced inflammation, and induced a more favorable lipid profile, and reduced insulin resistance.^29^ Following dietary intervention, we observed no statistical difference between the groups in the amount of kilocalories consumed. The HFF and NAF groups had similar kilocalories consumed but had vastly different levels of steatosis. These data would suggest that it is not a reduction in the kilocalories contributing to the resolution of steatosis, but rather using alternate sources of saturated fat (e.g. beef tallow and coconut oil), reducing carbohydrates and the addition of fiber in the HFF that is responsible for this reduction. On the other hand, mice receiving the LFF diet did have a reduction in kilocalories, though this was not significant when compared to the NAF diet. Therefore, the complete reduction in steatosis observed in LFF-fed mice may due to both the reduction of fats and the overall reduction in energy consumption.

We observed that mice on NAF for 6 weeks had a reduction in Bacteroidetes and an increase in Firmicutes, similar to reports in the literature using other high fat or Western-style diets.^30^ We also observed an increase in Verrucomicrobia, which correlated with the length of time on NAF diet and less on weight. The observed increase in Verrucomicrobia between the 6 and 12 week NAF mice was also observed in mice fed the LFF diet and, to a lesser extent, mice fed the HFF diet. Verrucomicrobia, specifically *Akkermansia mucinophilia*, has recently been shown to be reduced in mice fed high-fat diets and has been inversely correlated with body weight in both mice and humans.^31^ In this present study, Verrucomicrobia does not significantly correlate with body weight or liver histology since mice fed NAF for 12 weeks had the second highest abundance of this phyla. These data may indicate that the abundance of a single species is not enough to reverse weight gain. Instead, it may be a combination of microbes that together produce a metabolic signature that can help reduce steatosis and reverse obesity. In addition to Verrucomicrobia, LFF mice had an increase in *Bifidobacteriaceae*. Reports in the literature have shown that probiotic administration of *Bifidobacterium animalis* increases the abundance of *Akkermansia*.^32^ Therefore, it is possible that the dramatic effects in weight loss and the reversal of liver pathology in the LFF mice may be due to a symbiotic relationship between *Akkermansia* and *Bifidobacteriaceae*. This may explain why the LFF-MT failed to provide protection which had very low abundance of Actinobacteria. Overall these data suggest Verrucomicrobia alone is not enough to reverse or halt the progression of NAFLD but may act in concert with other commensals to impact disease.

The gut microbiota of NAF mice was dominated by *Erysipelotrichaceae* which was reduced in mice receiving either of the intervention diets. Mice that received HFF had increases in *Peptostreptococcaceae, Staphylococcaceae* and other families within the Bacilli class, while *Erysipelotrichaceae* was reduced. Unlike its HFF counterparts, mice switched to LFF had significant increases in Actinobacteria, Bacteroidetes and Verrucomicrobia with a significant decrease in Firmicutes including *Erysipelotrichaceae*. These results reflect recent observations in NAFLD patients in which the presence of *Erysipelotrichaceae* is associated with steatosis.^33^ There is also mounting evidence implicating a role for *Erysipelotrichaceae* in metabolic disorders and correlations between the levels of *Erysipelotrichaceae* and host cholesterol metabolites.^34^ The relationship between *Erysipelotrichaceae* and cholesterol is intriguing, especially as cholesterol was supplemented in our NAFLD-inducing diet and low in both HFF and LFF diets which had less *Erysipelotrichaceae*.

The major difference in the three diets was the fatty acid composition. The NAFLD-inducing diet had an equal ratio of saturated fatty acid (SFA) and monounsaturated fatty acids (MUFA) but had a third less polyunsaturated fatty acids (PUFA) with nearly 20 times more omega-6 than omega-3. The HFF diet had nearly 2.5 times more SFA than MUFA, however the sources of the SFA used for the diets were different (i.e. beef tallow and coconut oil versus anhydrous milkfat, lard and vegetable shortening). Unlike the NAFLD-inducing SFA that are comprised mostly of palmitic and stearic acid, the SFA in the HFF diet was enriched for lauric acid. Lauric acid has been shown to raise total cholesterol levels, specifically increasing high-density lipoproteins which has been shown to have health benefits.^35^ Despite the high-fat content, the HFF diet was able to reverse steatosis and progression of NAFLD. Though the role of medium chain fatty acid (MCFA) and medium chain triglycerides (MCT) seems controversial as MCT has been shown to cause steatosis,^36^ while other studies have shown they reduce steatosis and hepatic injury by upregulating fatty acid liver oxidation.^37,38^ Although MCFAs are associated with decreasing the ratio of Firmicutes to Bacteroidetes leading to a reduction in steatosis, we did not observe such changes in the HFF mice. In contrast, the LFF diet had over 4 times less fat than both the NAFLD-inducing and the HFF diets and the fat present was mainly enriched in omega-3 PUFAs. A recent randomized clinical trial administering omega-3 to individuals saw increases in *Bifidobacterium*^39^ similar to what we observed in the LFF mice.

Dietary modifications impact both the physiology of the host and the microbiota. In order to understand how dietary shifts in microbial composition impacted the progression of NAFLD, we performed microbiota transplants. Mice were administered cecal contents from donors fed either of the intervention diets or from those that remained on NAF throughout the experiment. Unlike the intervention diets, microbiota transplantation showed no improvement in histology, steatosis, AST or ALT levels, or liver cytokines. In fact, mice receiving the microbiota from LFF mice had extensive pericellular fibrosis and necro-inflammatory foci. Our data is in contrast to recent studies that have shown slight beneficial effects of fecal microbiota transplant (FMT) in treating metabolic disease in human and animal studies.^40-42^ While several clinical reviews propose microbiota transplants, only one has tried FMTs in a diet-induced NAFLD mouse model. Zhou and colleagues fed mice a high-fat diet and gave daily FMT from healthy, untreated mice for 8 weeks. The mice receiving FMTs had marked improvements in hepatic steatosis, lobular inflammation and hepatocyte ballooning, as well as a reduction in body weight, epididymal fat and serum levels of ALT.^42^ In contrast, our study found that microbiota transplantation was not as effective. Obviously, the frequency of FMT and the diets of the donor mice differ greatly between the two studies. Additionally, our study did not evaluate the impact of microbiota transplants derived from donors on standard mouse chow. Instead, our study aimed to identify clinically relevant diets and their components that exacerbated or ameliorated NAFLD. While the study by Zhou et al. demonstrates the importance of restoring a ‘physiological’ gut microbiome, our current study shows that microbiota transplants may not be as advantageous as dietary modifications. These contrasting results highlight the critical need for clinical validation of FMTs.

Altogether, these data demonstrate that altering the diet composition at the macronutrient level can reverse NAFLD. Specifically, we found that a diet high in saturated fats derived from products that have high medium chain fatty acids, high in fiber and high in omega-3 PUFAs or a diet low in fats but high in omega-3 PUFA were both able to reverse the progression of NAFLD. However, microbiota transplantation using cecal contents was unable to confer protection from NAFLD and, in some instances, lead to a more severe pathology. Our data suggest that microbial transplants to treat NAFLD may not be useful in the absence of continued dietary modifications and that more work must be done to understand the interaction between diet and the microbiome in the context of a given disease before FMTs can be exploited to treat disease.

## Materials & methods

### Experimental animals

Male C57BL/6 mice 3–4 weeks of age were purchased from Jackson Laboratory (Bar Harbor, Maine USA) and maintained at the University of Southern California (USC; Los Angeles, CA) animal facility under specific pathogen-free conditions. Ten mice per group (five groups: NC, NAF6, NAF, LFF and HFF) were used and the mice were housed in pairs or triads (4 cages for each group) for a total of 50 mice used in the intervention study. For the transplant study 3–9 mice were used per group (NAF-MT, LFF-MT, HFF-MT and NAF control) for a total of 21 mice. Together 71 mice were used in this study. Mice were fed indicated diets for 6 weeks or 12–14 weeks with sterile, distilled non-chlorinated and non-acidified water *ad libitum*. All animal experiments were performed following experimental review and approval by the Institutional Biosafety Committee and the Institutional Animal Care and Use Committee at the University of Southern California.

### Rodent diets

Experimental mice were fed a NAFLD-inducing (NAF) diet that contains 50% of kcal from fat, 20% sucrose, 10% fructose, and 1.25% cholesterol (TD. 150235, Teklad Diet, Envigo, Madison, Wisconsin USA www.envigo.com). After 6 weeks, some mice were examined for liver pathology, steatosis and fibrosis, (n = 5–8/group) one group was switched to a dietary intervention (n = 5–8/group), and an additional group was treated with antibiotic-water and given a fecal microbiota transplant (n = 6–9/group). Mice undergoing dietary intervention were immediately switched to a high-fat, high fiber (HFF) diet containing 66% of kcal from fat (primarily beef tallow and coconut oil) and increased non-digestible fibers or a low-fat, low-fiber (LFF) diet containing 20% of kcal from fat (primarily beef tallow and coconut oil) for 8 weeks. Control mice were fed the NAF diet for a total of 12–14 weeks.

### Microbiota transplant

For microbiota transplant (MT), mice on the NAF diet for 6 weeks were placed on antibiotic water containing 1 g/L ampicillin (Alfa Aesar), 1 g/L gentamicin (Amresco), 1 g/L neomycin (Sigma Aldrich), 1 g/L metronidazole (MP Biomedicals) and 0.5 g/L vancomycin (Alfa Aesar) for 1.5 weeks. After termination of the antibiotic water, mice received three oral gavages every other day of cecal contents that were collected from mice that were fed one of the intervention diets or had continued on a NAF diet. The donor cecums were collected from five mice per group and each cecal content was resuspended in 1 ml of sterile PBS. In order to make sure there was no bias, 500 ul of cecal solution from each mouse was pooled together. Recipient mice were given 100uL of cecal mixture by oral gavage using a 28 gauge, round tipped needle. MT mice were maintained on NAF diet and the experiment was terminated 2 weeks following the first MT gavage.

### Tissue collection

Stool was collected prior to dietary intervention and 6 weeks following dietary intervention. All other tissue samples were collected at the termination of the experiment. Blood was obtained via terminal cardiac puncture. Subcutaneous white adipose tissue from the right hind limb and the right-sided epididymal white adipose tissue were excised. Contents from the ileum were removed with forceps. The liver was isolated from its vascular attachments and gallbladder. All tissue samples were weighed, snap frozen in liquid nitrogen and stored at −80°C.

### Liver histology

Liver tissue fixed in 4% paraformaldehyde was transferred into 95% ethanol and processed at AML laboratories (Baltimore, Maryland). Histologic sections were stained with hematoxylin and eosin (H&E) and Masson trichrome stains to assess and grade NAFLD and degrees of fibrosis.

### NAFLD activity score (NAS) and fibrosis

H&E stained slides were evaluated for the percentage of hepatic steatosis, portal inflammation, lobular inflammation and hepatocyte ballooning. These slides were also assessed for the presence or absence of Mallory-Denk bodies, megamitochondria, lipogranuloma and glycogenated nuclei. The NAFLD activity score (NAS) was scored on cumulative scoring of steatosis, lobular activity and hepatocyte ballooning with maximum score of “8”.^43,44^ Steatosis was scored between “0” and “3” with less than 5% steatosis scored “0”, 5–55% scored “1”, 33–65% scored “2”, and greater than 66% scored “3”. Lobular activity was scored between “0” and “3” with no lobular necroinflammatory infiltrates given a score of “0”, 1–2 foci scored “1”, 2–4 foci scored “2” and more than 4 foci scored “3”. No hepatocyte ballooning scored “0”, occasional presence scored “1”, many presences scored of “2” was issued. Fibrosis was graded on Masson trichrome stains and scored from “0” to “4”. No fibrosis scored “0”, presence of either perisinusoidal or periportal fibrosis scored “1”, presence of both scored “2”, presence of bridging fibrosis scored “3” and frank cirrhosis scored “4”.

### Liver cytokine enzyme-linked immunosorbent assay (ELISA)

Liver tissue samples (100 mg) that were previously frozen at −80°C were thawed and homogenized using 50 uL garnet beads and 1 mL of phosphate-buffered saline supplemented with protease and phosphatase inhibitors (Pierce, Thermo Scientific) the Omni International Bead Ruptor 12. Homogenized liver tissue was analyzed for: tumor necrosis factor (TNF)-α, interleukin (IL)-10, IL-12p40, and IL-6 (BD Biosciences) by ELISA. TMB substrate (Dako, Carpinteria, CA) was used for detection and absorbance was read at optical density (OD) 450 nm with 570 nm correction.

### Microbiota sequencing, sequence curation and analysis

Microbiota samples were processed and sequenced at Research and Testing Laboratory (RTL; Lubbock, TX) based upon RTL protocols using MiSeq Illumina platform. Universal bacterial primers 515 F ‘GTGCCAGCMGCCGCGGTAA’ and 806 R ‘GGACTACHVGGGTWTCTAAT’ were used to amplify the variable regions V3-V4 of the 16 S rRNA genes. 16 s rRNA gene sequences were curated using mothur v.1.36.1,^45^ following the MiSEQ SOP. Briefly, sequences were denoised using a flowgram denoising algorithm,^46^ aligned to Silva 16 s rRNA sequence database^47^ and pre-clustered to allow up to a 2-bp difference between sequences.^48^ Chimeras were detected using UCHIME^49^ and were culled along with chloroplast and mitochondrial sequences. Sequences were then classified using the Ribosomal Database Project version 14 with a confidence score greater than 80%^50^ and phylotyped to the family level. Prior to any further data analysis, the number of sequences were normalized to 2000 reads per sample. Beta diversity was calculated using the Theta YC distance metric with the family-level data and visualized using principal coordinates analysis (PCoA).

### Statistical analysis

Data are shown as the mean ± standard error of the mean (SEM). Student T-tests, one-way ANOVAs and two-way ANOVAs with post-hoc tests were used to determine statistical significance between the dietary treatments as indicated in each figure. Graphpad Prism (San Diego, CA) was used for graphical and statistical analysis and *p* < .05 was considered statistically significant. To compare the NC and NAF groups, student’s unpaired t-test was used and 2-way ANOVA with Sidak’s multiple comparison was used for body weight change over time. To compare the NAF at 6 weeks with intervention diets HFF and LFF and compare among the microbiota transplant groups, one-way ANOVA with Dunnett’s multiple comparison was used and 2-way ANOVA with Sidak’s multiple comparison was used for body weight change over time. For the microbiome analyses, Pearson Correlation Coefficient was used to analyze the correlation between weight and certain bacterial families. 16 S sequencing data were evaluated for statistical significance using analysis of molecular variance (AMOVA) and homogeneity of molecular variance (HOMOVA) tests of PCoAs on mothur v.1.36.1.^26,45^ Phylogenetic Investigation of Communities by Reconstruction of Unobserved States (PICRUSt) (http://picrust.github.io/picrust/) was used to evaluate the metabolic components and further visualized and analyzed with Statistical Analysis of Metagenomic Profiles (STAMP) software (https://beikolab.cs.dal.ca/software/STAMP).

## Supplementary Material

Supplemental Material

## Abbreviations

ALT: alanine aminotransferase
AST: aspartate aminotransferase
GF: germ-free
HFF: high fat and fiber
IL: interleukin
LFF: low fat and fiber
NAFLD: nonalcoholic fatty liver disease
MT: microbiota transplant
NAF: NAFLD-inducing diet
NAF6: NAFLD-inducing diet taken at 6 weeks
NAS: NAFLD activity score.
