# Supplementary material for: Modifying macronutrients is superior to microbiome transplantation in treating nonalcoholic fatty liver disease
Source: Gut Microbes. 2020 Aug 20;12(1):1792256. doi: 10.1080/19490976.2020.1792256 (PMC7524401; doi:10.1080/19490976.2020.1792256)
Supplement: Supplemental Material [file KGMI_A_1792256_SM4068.docx]

Supplemental Table 1: Dietary Breakdown

| **g / kg** | **NAF** | **HFF** | **LFF** |
| --- | --- | --- | --- |
|  | **TD.150235** | **TD.150587** | **TD.150588** |
| ME, kCal/g | 4.89 | 3.98 | 3.36 |
| Casein | 160 | 130 | 130 |
| L-Cystine | 1.5 | 3 | 3 |
| Mineral Mix, AIN-93M-MX | 45 | 45 | 35 |
| Vitamin Mix, AIN-93-VX | 20 | 20 | 10 |
| DL-Methionine, FG (99%) | - | 2 | 2 |
| Egg White Solids, spray-dried | - | 36 | 36 |
| Egg Yolk Powder | - | 10 | 10 |
| Fish Meal, menhaden | - | 5 | 5 |
| High Amylose Corn Starch | - | 50 | 482 |
| Sucrose | 200 | 116.936 | 168.482 |
| Fructose | 100 | - | - |
| Corn Starch | 44.96 | - | - |
| Maltodextrin | 100 | - | - |
| Anhydrous Milkfat | 94 | - | - |
| Vegetable Shortening, hydrogenated | 105 | - | - |
| Palm Oil | 74 | - | - |
| Coconut Oil | - | 122 | 2 |
| Beef Tallow | - | 89 | 2 |
| Fish Oil | - | 32 | 25 |
| Lard | - | 10 | 2 |
| Flaxseed Oil | - | 33 | 33 |
| Cholesterol | 12.5 | - | - |
| Cellulose | - | 290 | 50 |
| Choline Bitartrate | 2 | 2 | 2 |
| Biotin | - | 0.004 | 0.004 |
| TBHQ, antioxidate | 0.04 | 0.06 | 0.14 |
| Potassium Phosphate, dibasic | - | 4 | 2.5 |

Supplemental Table 2: Relationship among *Erysipelotrichaceae* and *Verrucomicrobiaceae*

| **% Erysipelotrichaceae vs % Verrucomicrobiaceae** | | |  |  |
| --- | --- | --- | --- | --- |
| **Pearson r** | p-value | 0.0008 | *** |  |
|  |  |  |  |  |
|  |  |  |  |  |
|  | **X vs. NAF6** | **X vs. NAF** | **X vs. HFF** | **X vs. LFF** |
| **Pearson r** |  |  |  |  |
| r | -0.9499 | -0.961 | 0.8451 | -0.7803 |
| 95% confidence interval | -0.9911 to -0.7418 | -0.9975 to -0.5177 | -0.1463 to 0.9895 | -0.9658 to -0.06604 |
| R squared | 0.9024 | 0.9236 | 0.7141 | 0.6089 |
|  |  |  |  |  |
| P value |  |  |  |  |
| P (two-tailed) | 0.0003 | 0.0092 | 0.0715 | 0.0385 |
| P value summary | *** | ** | ns | * |
| Significant? (alpha = 0.05) | Yes | Yes | No | Yes |
|  |  |  |  |  |
| Number of XY Pairs | 8 | 5 | 5 | 7 |

Supplemental Table 3: Relationship among bacterial families and weight change

| **% *Erysipelotrichaceae* vs weight change** | |  |  |
| --- | --- | --- | --- |
| **Pearson r** | p-value | 0.0035 | ** |
|  |  |  |  |
|  | **X vs. NAF** | **X vs. HFF** | **X vs. LFF** |
| r | -0.7709 | 0.6661 | 0.4061 |
| 95% confidence interval | -0.9839 to 0.3482 | -0.5242 to 0.9752 | -0.4998 to 0.8877 |
| R squared | 0.5943 | 0.4437 | 0.165 |
| P value |  |  |  |
| P (two-tailed) | 0.127 | 0.2196 | 0.3659 |
| P value summary | ns | ns | ns |
|  |  |  |  |
| **% *Verrucomicrobiaceae* vs weight change** | |  |  |
| **Pearson r** | p-value | 0.2494 | ns |
|  |  |  |  |
|  | **X vs. NAF** | **X vs. HFF** | **X vs. LFF** |
| r | -0.839 | 0.8183 | -0.1127 |
| 95% confidence interval | -0.9891 to 0.1666 | -0.2302 to 0.9876 | -0.7980 to 0.6997 |
| R squared | 0.7039 | 0.6695 | 0.01271 |
| P value |  |  |  |
| P (two-tailed) | 0.0757 | 0.0904 | 0.8098 |
| P value summary | ns | ns | ns |
|  |  |  |  |
| **% *Bifidobactereaceae* vs weight change** | |  |  |
| **Pearson r** | p-value | 0.0271 | * |
|  |  |  |  |
|  | **X vs. NAF** | **X vs. HFF** | **X vs. LFF** |
| r | 0.6264 | 0.6993 | -0.1754 |
| 95% confidence interval | -0.5719 to 0.9717 | -0.4777 to 0.9781 | -0.8201 to 0.6656 |
| R squared | 0.3924 | 0.489 | 0.03077 |
| P value |  |  |  |
| P (two-tailed) | 0.2582 | 0.1888 | 0.7068 |
| P value summary | ns | ns | ns |

NC NAFLD-diet 14 weeks


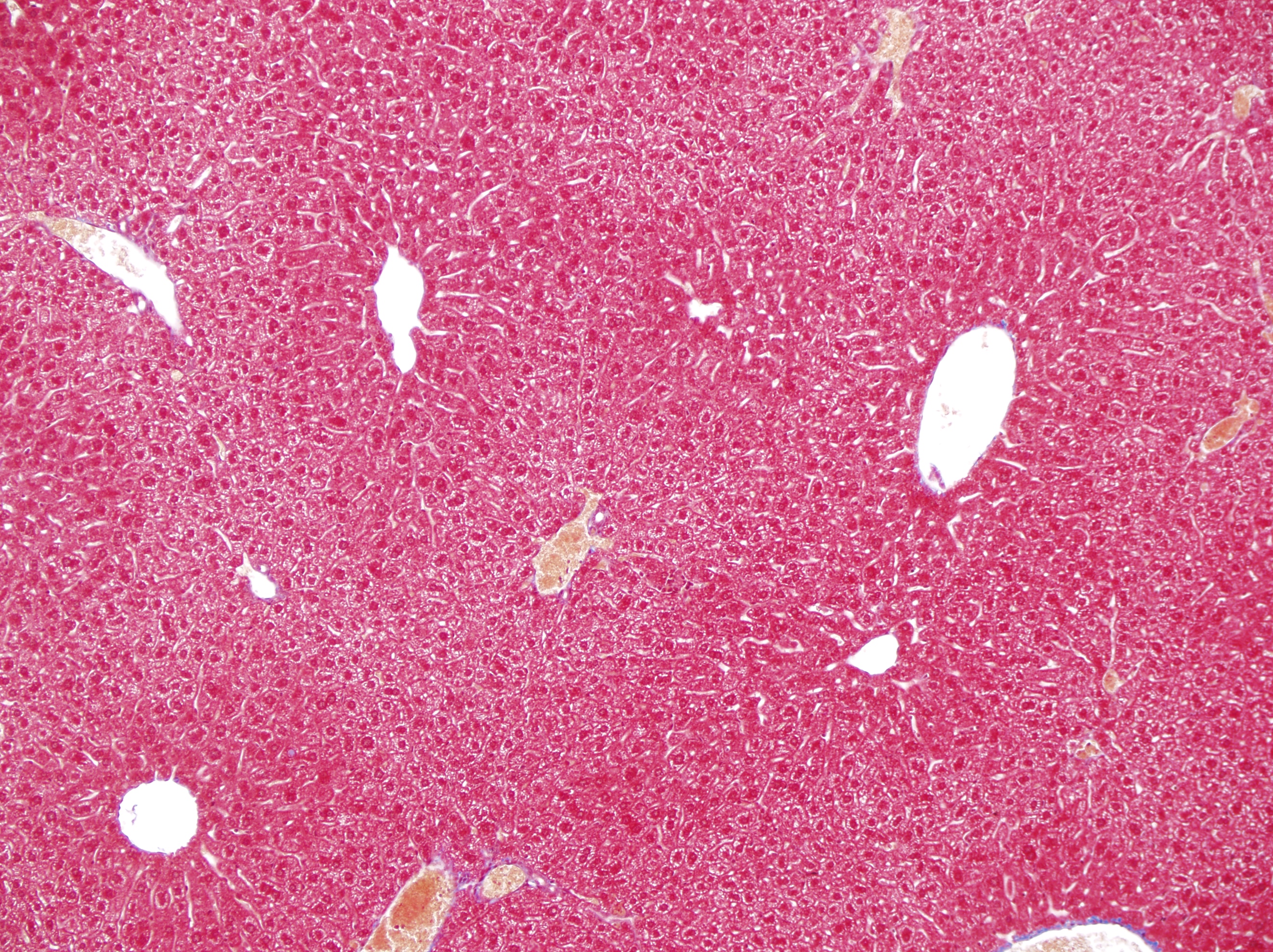

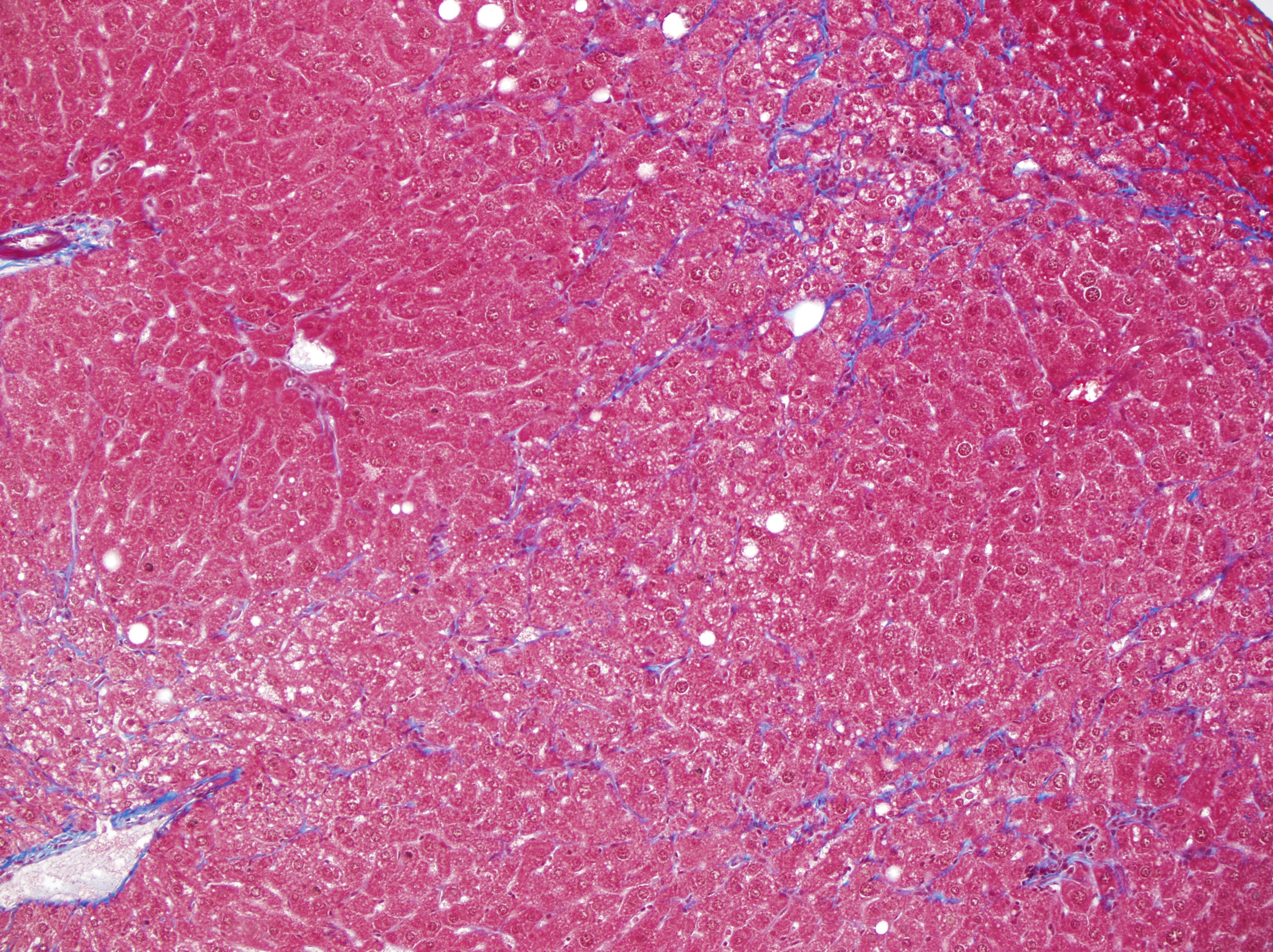


Supplementary Figure 1

**Fibrosis in liver observed after 14 weeks on the NAFLD diet.** Representative images of trichrome stained liver sections from mice maintained on a normal rodent chow (NC) or fed a NAFLD-inducing diet.


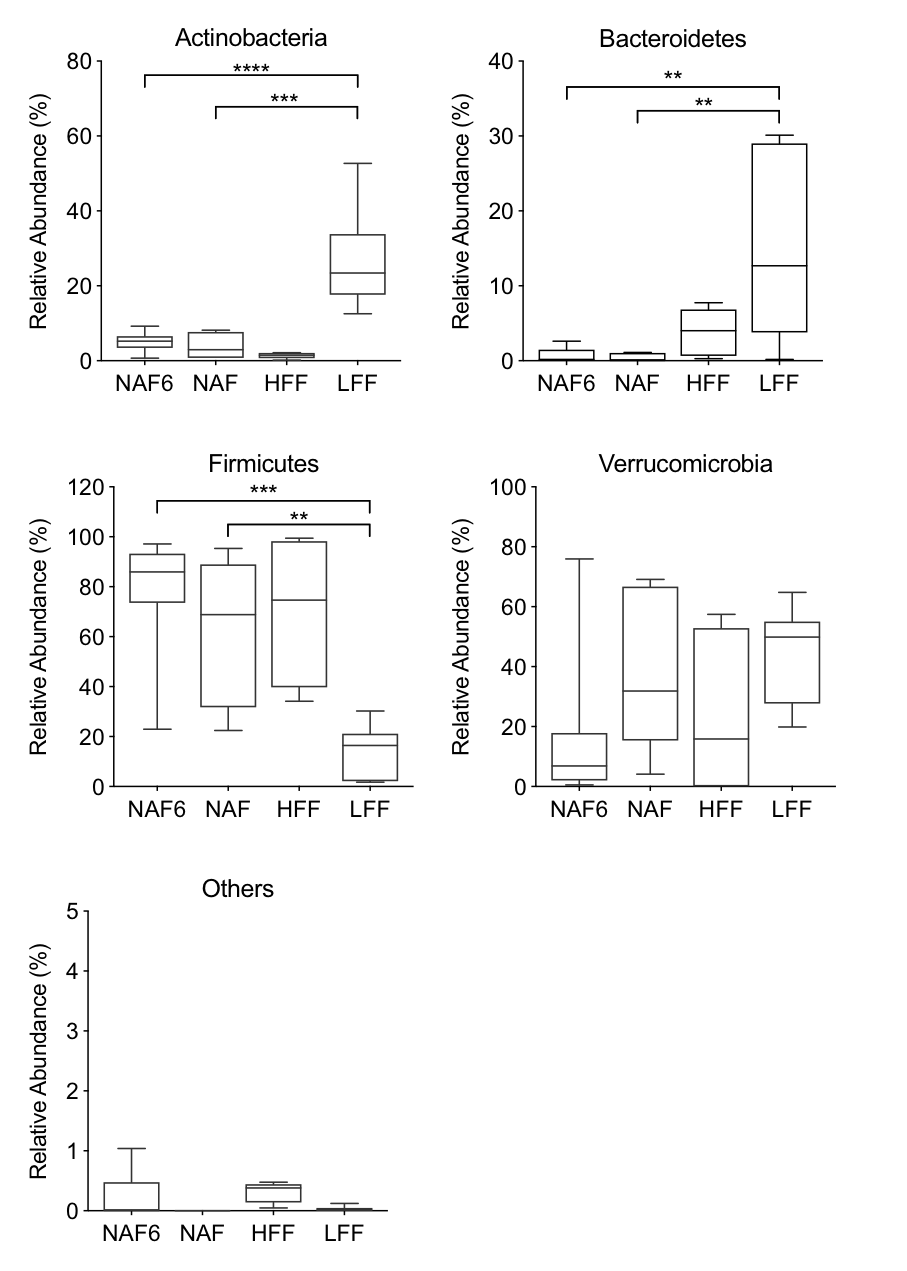


Supplemental Figure 2.

Microbiota sampling of fecal contents of mice in treatment groups NAF6, NAF, HFF and LFF. Relative abundance of major phylum-level commensals. Data is the mean ± SEM of 2 independent experiments, n=5-9mice/group. One-way ANOVA, Dunnett’s multiple comparisons; **, p<0.01; ***, p<0.005; and ****, p<0.001.

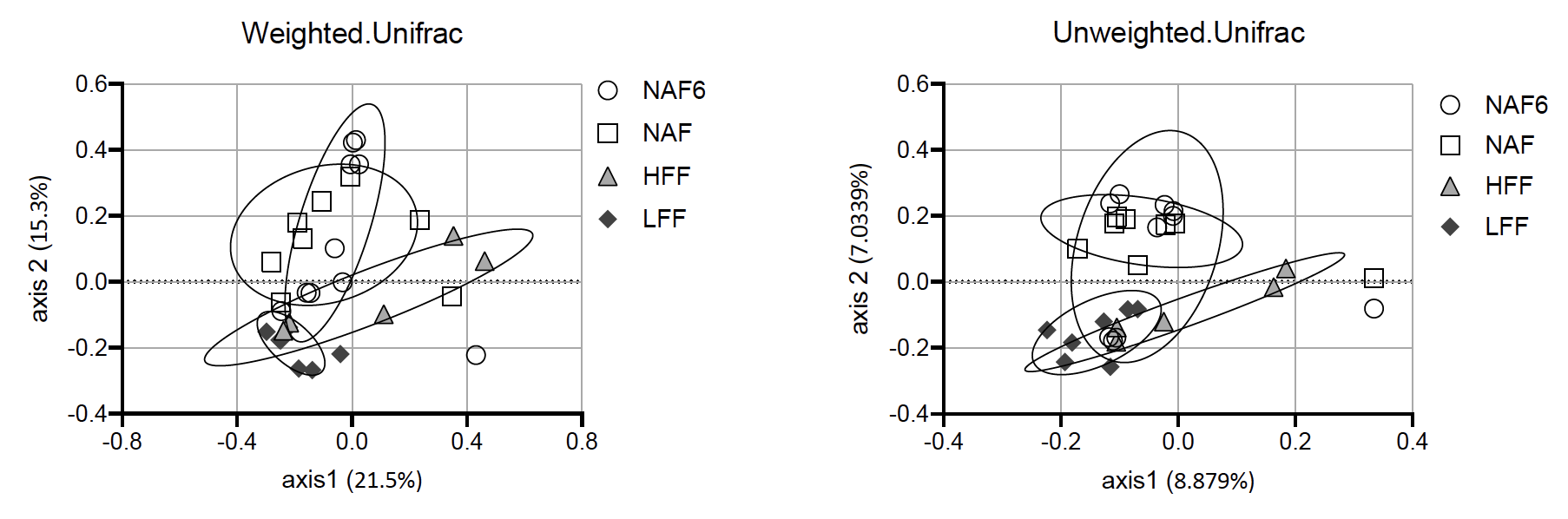


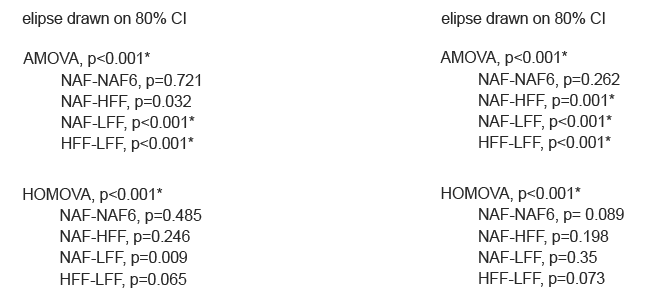


Supplemental Figure 3

Microbiota sampling of ileal contents of mice in treatment groups NAF6, NAF, HFF and LFF. (A) Phylum-level abundance and Heatmap of family-level abundance with families >1% abundance. Data is the mean ± SEM of 2 independent experiments, n=4-8mice/group. PCoA of β−diversity as calculated using weighted and unweighted Unifrac distances.

A.


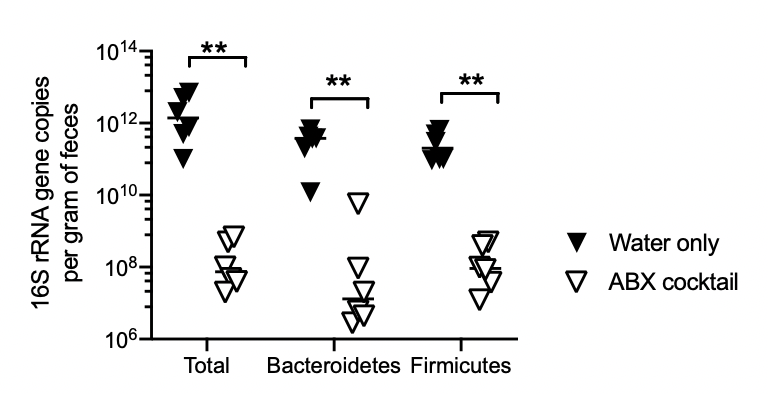


B.

Supplemental Figure 4.

(A) Gene copy number of bacteria in fecal samples after 10 days of ABX cocktail (AVNMG). To determine approximate bacterial numbers, a standard curve was generated using plasmids containing a cloned 16s rRNA gene from a representative bacterial species. (B) Weight of white adipose tissue as percentage of body weight of mice following microbiota transplant using cecal contents of donors previously on intervention diets. NAF open circles represent NAF MT while closed circles represent NAF control mice. Data is the mean ± SEM of 2 independent experiments, n=3-9mice/group.

‘

Supplemental Figure 5

Mean relative abundance of major phylum-level commensals from the fecal contents of mice receiving microbial transplants n=3-9 mice/group of mice


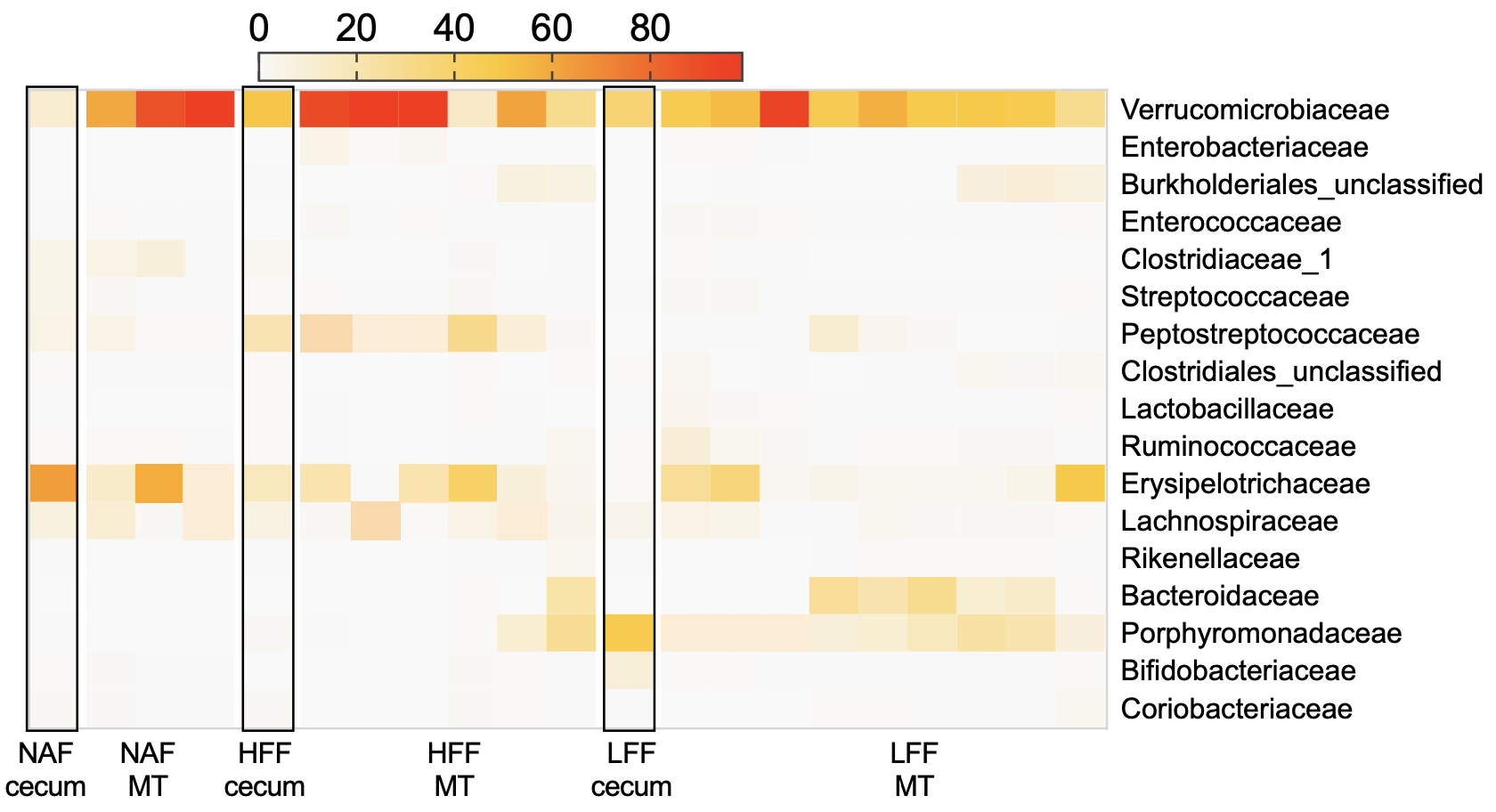


Supplemental Figure 6

Supplemental figure 6

Fecal microbiota of mice post microbiota transplant was sampled. Heatmap of family-level abundance with families >1% abundance. Data is from 2 independent experiments, n=3-9mice/group.
